# Functional analysis of *HOXD9 *in human gliomas and glioma cancer stem cells

**DOI:** 10.1186/1476-4598-10-60

**Published:** 2011-05-22

**Authors:** Masanao Tabuse, Shigeki Ohta, Yohei Ohashi, Raita Fukaya, Aya Misawa, Kazunari Yoshida, Takeshi Kawase, Hideyuki Saya, Cécile Thirant, Hérve Chneiweiss, Yumi Matsuzaki, Hideyuki Okano, Yutaka Kawakami, Masahiro Toda

**Affiliations:** 1 Neuroimmunology Research Group, Keio University School of Medicine, 35 Shinanomachi, Shinjuku-ku, Tokyo 160-8582, Japan; 2 Department of Neurosurgery, Keio University School of Medicine, 35 Shinanomachi, Shinjuku-ku, Tokyo 160-8582, Japan; 3 Division of Cellular Signaling, Institute for Advanced Medical Research, Keio University School of Medicine, 35 Shinanomachi, Shinjuku-ku, Tokyo 160-8582, Japan; 4 Division of Gene Regulation, Institute for Advanced Medical Research of Keio University School of Medicine, 35 Shinanomachi, Shinjuku-ku, Tokyo 160-8582, Japan; 5 Team Glial Plasticity Inserm UMR 894, University Paris Descartes, 75014 Paris, France; 6 Division of Physiology, Keio University School of Medicine, 35 Shinanomachi, Shinjuku-ku, Tokyo 160-8582, Japan

## Abstract

**Background:**

*HOX *genes encode a family of homeodomain-containing transcription factors involved in the determination of cell fate and identity during embryonic development. They also behave as oncogenes in some malignancies.

**Results:**

In this study, we found high expression of the *HOXD9 *gene transcript in glioma cell lines and human glioma tissues by quantitative real-time PCR. Using immunohistochemistry, we observed HOXD9 protein expression in human brain tumor tissues, including astrocytomas and glioblastomas. To investigate the role of *HOXD9 *in gliomas, we silenced its expression in the glioma cell line U87 using *HOXD9*-specific siRNA, and observed decreased cell proliferation, cell cycle arrest, and induction of apoptosis. It was suggested that *HOXD9 *contributes to both cell proliferation and/or cell survival. The *HOXD9 *gene was highly expressed in a side population (SP) of SK-MG-1 cells that was previously identified as an enriched-cell fraction of glioma cancer stem-like cells. *HOXD9 *siRNA treatment of SK-MG-1 SP cells resulted in reduced cell proliferation. Finally, we cultured human glioma cancer stem cells (GCSCs) from patient specimens found with high expression of *HOXD9 *in GCSCs compared with normal astrocyte cells and neural stem/progenitor cells (NSPCs).

**Conclusions:**

Our results suggest that *HOXD9 *may be a novel marker of GCSCs and cell proliferation and/or survival factor in gliomas and glioma cancer stem-like cells, and a potential therapeutic target.

## Background

Gliomas, especially glioblastomas (GBMs), are the most malignant primary brain tumors[1]. The median survival of a patient with GBMs is 15 months, and this has improved little by temozolomide treatment[2]. GBMs have a high rate of cellular proliferation and a marked propensity to invade remote brain structures. Such aggressive and invasive growth is the hallmark feature that gives rise to their high morbidity and mortality. A better understanding of the mechanisms underlying the initiation and progression of GBMs at the molecular and cellular levels will open up new opportunities to develop therapeutic strategies.

The growth of many tumors depends on a subset of tumor cells with an extensive capacity for self-renewal, called either cancer stem cells (CSCs) or tumor-initiating cells[3]. Several studies report the presence of CSCs in gliomas[4,5]. In glioma CSC (GCSC), the expression of some neural stem markers, such as *SOX2 *and *Musashi-1*, has been reported[6,7]. In addition, some markers including CD133[8] and SSEA-1 (CD15)[9] have been evaluated as GCSC enrichment markers; however, several studies show their limitations as specific markers[10,11].

Homeobox proteins are master regulators of development and control many cellular processes, including proliferation, apoptosis, cell shape, and cell migration. Homeobox proteins belong to a superfamily, and are encoded by a number of genes, such as *SIX*, *MSX*, *PAX*, *LIM*, and *HOX*[12]. Among Homeobox proteins, *HOX *genes encode transcription factors that act as critical regulators of growth and differentiation, control of cell identity, cellular communication, cell cycle progression, hematopoiesis, and apoptosis in addition to the control of axial patterning during embryogenesis. In humans, 39 *HOX *genes have been identified and partly or fully redundant functions of *HOX *genes have also been known[13].

*HOX *genes have been reported to be misexpressed in many tumors including lung carcinoma, neuroblastoma, ovarian carcinoma, cervical carcinoma, prostate carcinoma, breast carcinoma, and leukaemia[14]. In addition, epigenetic control of *HOX *genes in development and diseases have been shown in many studies[15]. In a previous study, we performed restriction landmark genomic scanning (RLGS) with a CpG methylation-sensitive enzyme to identify CpG islands of genes that are differentially methylated in human glioma cells compared with normal lymphocytes to find epigenetically controlled genes in glioma[16], and identified 12 genes that seem to be regulated by epigenetic gene modification. One of the identified genes was *HOXD9*[16]. *HOXD9 *is critical for embryonic segmentation[17] and limb bud patterning[18] during development, but its biological function in human adult tissues has been elusive.

Recently, it was reported that *HOXD *genes were expressed in neoplastic astrocytes[19] and pediatric low-grade gliomas[20]. However much less is known about the function of *HOXD *genes especially in gliomas. In this study, we analyzed the expression and function of *HOXD9 *in human gliomas and found high expression of *HOXD9 *in GCSCs. *HOXD9 *contributes to cell proliferation and/or survival in glioma cells and glioma cancer stem-like cells. Thus, *HOXD9 *may be a new target for the treatment of gliomas based on GCSC population.

## Methods

### Tissue samples and cell lines

All tumor tissue specimens were obtained from patients with glioma who underwent surgery at the Department of Neurosurgery, Keio University School of Medicine and the Department of Neurosurgery, Sainte Anne Hospital, Medical School of Paris Descartes University. Tumors obtained from surgical cases were classified according to the World Health Organization (WHO) criteria[21] as follows: glioblastoma (WHO grade IV), anaplastic astrocytoma (WHO grade III), or diffuse astrocytoma (WHO grade II). Written informed consent was obtained from all patients in the study, which was conducted in accordance with the Institutional Review Board guidelines of Keio University or Paris Descartes University. Human glioma cell lines (U87, SK-MG-1, KNS42, KNS81) were obtained from the American Type Culture Collection (Manassas, VA) and the Japanese Collection of Research Bioresources (Osaka, Japan), and maintained in Dulbecco's modified Eagle's medium (Wako, Tokyo, Japan) supplemented with 10% fetal bovine serum (FBS) and antibiotics (50 IU/ml benzyl penicillin G potassium and 100 μg/ml streptomycin sulfate; Meiji, Tokyo, Japan). Side population (SP) and non-SP SK-MG-1 cells were isolated using a flow cytometer (EPIC Altra; Beckman Coulter, Tokyo, Japan) as previously described[22]. Human neural stem/progenitor cells (NSPCs) were cultured as neurospheres (NSPs) in neurosphere culture medium consisting of Neurobasal Medium (Invitrogen, Carlsbad, CA) supplemented with human recombinant (hr) EGF (20 ng/mL; Peprotech, Rocky Hills, NY), hrFGF2 (10 ng/mL; Peprotech), hrLIF (10 ng/mL; Millipore-Japan, Tokyo, Japan), heparin (5 μg/ml; Sigma, St. Louis, MO) and B27 (Invitrogen) as previously described[23]. Primary astrocyte cells were purchased from Takara Bio (Tokyo, Japan). GCSC lines were established from human glioma tissue specimens as described in a previous study[10].

### RNA extraction and quantitative (q) RT-PCR

Total RNA was isolated from human glioma tissues and cell lines using Trizol (Invitrogen). Total RNA from normal tissues was purchased from Clontech (Palo Alto, CA). Synthesis of cDNA was performed using 1 μg of total RNA using Reverse transcriptase XL (AMV) or PrimeScript RT Master Mix (Takara Bio). The primers were designed as follows: for *HOXD9*, forward primer, 5'-GAGGAGGAGAAGCAGCATTC-3', reverse primer, 5'-TTCTCCAGCTCAAGCGTCTG-3'; for *SOX2*, forward primer, 5'-ATGGACAGTTACGCGCACA-3', reverse primer, 5'-TGCGAGTAGGACATGCTGTA-3'; for *BCL-2*, forward primer, 5'-AGGATTGTGGCCTTCTTTGAGT-3', reverse primer, 5'-GCCGGTTCAGGTACTCAGTCAT-3'; for *TRAIL *forward primer, 5'-CGTGTACTTTACCAACGAGCTGA-3', reverse primer, 5'-ACGGAGTTGCCACTTGACTTG-3'; and for *GAPDH*, forward primer, 5'-TGAACGGGAAGCTCACTGG-3', reverse primer, 5'-TCCACCACCTGTTGCTGTA-3'. We designed intron-spanning primers to amplify *HOXD9 *and *SOX2*. For *BCL-2 *and *TRAIL*, respectively, previously tested and optimized primer sets were used as described in Brown (2007)[24] and Williams (2003) [25]. Quantitative RT-PCR analysis was performed with a fluorescent dye, SYBR Green (Applied Biosystems, Foster City, CA), using the ABI prism 7900 HT Sequence Detection System (Applied Biosystems) as previously described[23]. The PCR parameters were as follows: 10 min at 95°C, then 40 cycles of denaturation at 95°C for 1 min, annealing at 60°C for 1 min, and extension at 72°C for 1 min. The relative gene expression level was normalized to that of *GAPDH *in each sample and calculated as the threshold cycle (CT) value in each sample divided by the CT value in each reference. The CT value is defined as the value obtained in the PCR cycle when the fluorescence signal increases above the background threshold.

### Microarray procedure and data processing

Approximately 10^6 ^U87 cells were used for total RNA extraction using the RNeasy mini kit (Qiagen, Valencia, CA) according to the manufacturer's instructions. RNA quality was verified with the Bioanalyzer System (Agilent Technologies, Palo Alto, CA) using RNA Nano Chips. RNA (1.5 μg) was processed for hybridization on the Genechip Human Genome U133 Plus 2.0 Expression array (Affymetrix, Santa Clara, CA), which contains over 54,000 probe sets for analyzing the expression level of over 47,000 transcripts and variants, including 38,500 well-characterized human genes. Processing was done according to the manufacturer's recommendations. Except when indicated, all genomic and transcript analysis was carried out using GeneSpring software 7.3.1 (Agilent Technologies). Microarray data were deposited at the NCBI Gene Expression Omnibus (GEO: GSE28618).

GEO data sets (http://www.ncbi.nlm.nih.gov/gds/) were used to analyze mRNA expression microarray data from several brain tumors and normal brain. The following samples were subjected to analysis: normal brain (65 normal human brain tissue samples from ten post-mortem donors, Roth et al., 2006[26], GSE3526); medulloblastoma (62 samples, Kool et al., 2008[27], GSE10327); oligodendroglioma grade II and III, astrocytoma grade II and III, and glioblastoma grade IV (157 samples, Sun L et al., 2006[28], GDS1962). Comparisons were made using the method of Kool et al[27]. The gene expression level of *HOXD9 *in brain tumors was compared to the expression in normal brain. Statistical values calculated normalized data from MAS 5 algorithm by the Wilcoxon signed rank test.

### Bisulfate genomic sequencing

Genomic DNA was purified from each cell line using the Wizard SV Genomic DNA Purification Kit (Promega, Madison, WI). T cells were isolated from peripheral blood mononuclear cells using magnetic beads conjugated to anti-human CD3 (Miltenyi Biotech, Tokyo, Japan). Bisulfate conversion was performed using 0.5-0.7 μg of genomic DNA and the reagents provided in the Qiagen EpiTect Bisulfate kit (Qiagen). The converted DNA was amplified by PCR using the following primers: 5'-GAGGGGAGAATAGTTTTTTT-3' and 5'-CAAACCCAAATCCATATACCC-3'. The PCR products were subcloned into the pGEM-T Easy vector (Promega) and verified by sequencing.

### Plasmid construction and transfection

pCMV6-XL5-*HOXD9 *containing a human full-length cDNA was obtained from Origen Technologies, Inc (Rockville, MD) and subcloned into a pMX-Ig vector (gifted from Dr. T. Kitamura) to generate the pMX-*HOXD9*. 293T and U87 cells were transfected with a pMX-*HOXD9 *plasmid using FuGENE HD transfection regent (Roche) according to the manufacturer's protocol.

### Western blot analysis

Cell lysates were prepared using the RIPA buffer (25 mM Tris-HCl, 150 mM NaCl, 1% NP-40, 1% sodium deoxycholate, and 0.1% SDS; pH 7.6) containing protease inhibitors (Cocktail Tablet; Roche Diagnostics, Japan). Lysates were centrifuged at 14,000×*g *for 15 min at 4°C, and the protein concentration of each sample was determined with the Bio-Rad protein assay kit (Bio-Rad, Hercules, CA) with bovine serum albumin as a standard. Identical amounts of the proteins were electrophoresed in 4-10% SDS-PAGE gels and transferred to a nitrocellulose membrane. Blots were blocked with Blocking One™ (Nacalai, Kyoto, Japan) at RT for 60 min, and incubated with either a goat anti-HOXD9 antibody (1:500; Santa Cruz Biotechnology. Inc, CA) and a rabbit anti-GAPDH antibody (1:4,000; Santa Cruz Biotechnology, Inc) overnight at 4°C. After being washed three times in TBST (20 mM Tris-HCl, 150 mM NaCl, and 0.02% tween-20; pH 7.4), the blots were incubated with the secondary antibody conjugated with horseradish peroxidase (1:4,000, anti-rabbit and anti-mouse; Thermo Scientific, Tokyo) for 1 h at room temperature. Signals were detected with a SuperSignal West Femto Maximum Sensivity Subdstrate (Thermo Scientific) and exposed to Hyperfilm (GE Health Care Biosciences). The specificity of HOXD9 antibody was further confirmed by a peptide-absorption assay (Additional file 1, Figure S1), in which the antibody was preincubated with the corresponding peptide antigen by 1:5 weight ratio at 4°C and subjected to the analysis.

### Small interfering RNA (siRNA) and transient transfection

siRNA duplexes were designed, synthesized, annealed, and purified by RNAi Co., Ltd (Tokyo, Japan). The sequences of the human *HOXD9*-specific siRNAs were 5'-GAGUUCGCCUCGUGUAGUUUU-3' (*HOXD9 *siRNA-1) and 5'- CCACUACGGGAUUAAGCCUGA-3' (*HOXD9 *siRNA-2). As a control, we used a non-silencing siRNA with the sequence 5'-GUACCGCACGUCAUUCGUAUC-3'. U87 or SK-MG-1 SP cells (5-6 × 10^4 ^cells/ml) were seeded in triplicate in tissue culture dishes (24-well or 96-well) 24 h prior to siRNA transfection. Control or *HOXD9 *siRNAs were transfected at a concentration of 50 nM using Lipofectamine RNAiMAX (Invitrogen) according to the manufacturer's instructions.

### Cell proliferation assay

Following siRNA transfection, cells were harvested by trypsinization and the total number of cells was counted by trypan blue exclusion under a phase-contrast microscope. A cell viability assay was performed using the Cell Titer-Glo Luminescent Cell Viability Assay kit (Promega) according to the manufacturer's protocol, using a luminometer (Wallac ARVO 1420 multilabel counter; WALLAC OY, Truku, Finland). Cell cycle analysis for live cells was performed using flow cytometry. siRNA-treated cells were stained with Vybrant DyeCycle Violet Stain (Invitrogen) for 30 min at 37°C according to the manufacturer's protocol and then subjected to flow cytometry (Gallios; Beckman Coulter). Raw data were analyzed using Multicycle for Windows (Beckman Coulter).

### Colony formation assay

48 h after siRNA transfection, cells (2 × 10^4 ^cells) were mixed with 2 ml of culture medium containing 0.4% agar and 10% FCS and then plated on 2 ml of the bottom layer containing 0.6% agar with 10% FCS in each well of a 6-well plate. Each experiment was performed in triplicate. After 3 weeks culture, colonies were counted after staining with MTT 3-(4,5-dimethyl-2-thiazolyl)-2,5-diphenyl-2H-tetrazolium bromide.

### Apoptosis assay

Caspase 3/7 activity in siRNA-transfected cells was measured using the Caspase-Glo 3/7 assay kit (Promega) according to the manufacturer's instructions. U87 cells were plated in 96-well plates at a seeding density of 6 × 10^3 ^cells/well, and caspase activity was assayed 48 h after siRNA transfection. To differentiate between apoptotic and necrotic cell death, siRNA-transfected cells (1 × 10^5 ^cells/ml) were stained with 5 μl Annexin-V antibody and 5 μl 7-AAD using an Annexin V-FITC/7-AAD-staining kit (Beckman Coulter), placed on ice for 10 min in the dark, and then analyzed by flow cytometry(EPICS XL; Beckman Coulter).

### Time-lapse cell imaging

Cells were transfected with control siRNA, *HOXD9 *siRNA-1 and *HOXD9 *siRNA-2. 24 h after transfection, 10,000 cells were plated on 24-well glass plates (IWAKI, Tokyo, Japan) and imaged using a NIKON TE2000-E microscope equipped with ×200 magnification lens (Nikon, Tokyo, Japan). The microscope stage was enclosed by a plastic air curtain and heated to 37°C. Time-lapse recordings were taken every 5 minutes for 96 h and analyzed using the image analysis software MetaMorph (Molecular Devices, Downingtown, PA). The cell division time of cells transfected with siRNAs was determined using the time-lapse images.

### Immunohistochemical staining

Paraffin-embedded tissue sections (5 μm) were deparaffinized in xylene and rehydrated. The sections were treated with a heat-based antigen retrieval method using a citrate solution (pH 6.0, 10 mM). Nonspecific binding of antibodies was blocked by incubation in 5% rabbit serum in 0.01 M PBS for 30 min. The slides were then incubated with goat anti-human HOXD9 polyclonal antibody (1:100; Santa Cruz Biotechnology, Inc., Santa Cruz, CA) diluted with 0.02% BSA in 0.01 M PBS overnight at 4°C in a humidified box. The slides were then incubated with a secondary antibody (Universal Immuno-peroxidase Polymer, Anti-goat; Histofine Simple Stain MAX PO (G), Nichirei Corporation, Tokyo, Japan) for 30 min at 37°C, and horseradish peroxidase labeling was visualized using 3,3'-diamonobenzidene (DAB). The sections were then lightly counterstained with hematoxylin. Each step was followed by three washes in PBS. To evaluate the proliferation of tumor cells, the same sections were stained with the monoclonal antibody MIB-1 (1:200; DAKO Japan, Kyoto, Japan) that recognizes the Ki-67 protein. The proliferating cell indexes were analyzed for >1000 tumor cells in more than three areas expressing the highest number of immunopositive nuclei. The analysis was performed using a digital camera (DXM-1200) attached to a microscope (Nikon) and ACT-1 software (Nikon). Immunohistochemical staining was performed in 29 glioma cases (5 diffuse astrocytomas, 11 anaplastic astrocytomas and 13 glioblastomas).

### Statistical analysis

The results are presented as mean values ± S.D. Data were analyzed using a Student's *t *test with *P *< 0.05 considered statistically significant.

## Results

### Analysis of *HOXD9 *gene expression in gliomas

**Figure 1:**
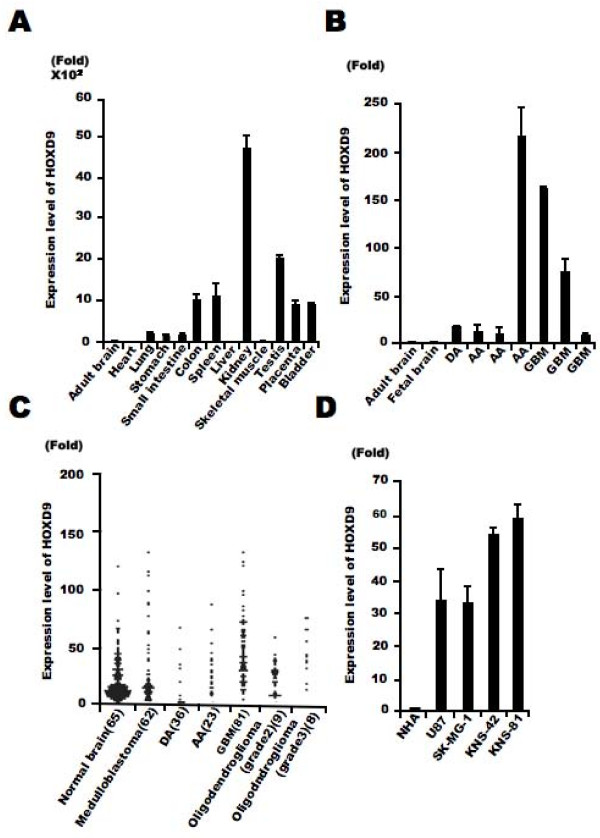
***HOXD9 *expression in gliomas**. (A) Analysis of *HOXD9 *gene expression in human normal tissues by qRT-PCR. (B) Analysis of *HOXD9 *gene expression in gliomas (diffuse astrocytoma [DA], anaplastic astrocytoma [AA], and glioblastoma multiform [GBM]) by qRT-PCR. (A), (B), Data were normalized using the results raised with the normal adult brain as reference. The graphs show the average of two independent experiments. Error bars indicate ± S.D. (C) Expression levels of *HOXD9 *in normal brain and brain tumors. Data were normalized using the results raised with the normal brain as reference. Gene expression and clinical outcome data were obtained from Gene Expression Omnibus (GEO) data sets at NCBI (GSE3526, GSE10327, GDS1962). (D) Analysis of *HOXD9 *gene expression in glioma cells and normal human astrocytes (NHA). For the graphs, the data are complied from three independent experiments. Error bars indicate ± S.D.

To examine the expression level of *HOXD9 *mRNA in tissues, we firstly performed qRT-PCR analysis in normal human tissues using intron-spanning gene-specific primers. *HOXD9 *was highly expressed in normal colon, spleen, kidney, testis, placenta, and bladder but poorly in the brain (Figure 1A). Next, we performed qRT-PCR analysis in glioma tissues obtained from our university hospital, all seven samples showed a >9-fold higher expression as compared with both normal fetal and adult brain samples (Figure 1B). This led us to investigate *HOXD9 *expression throught the published microarray databases. Bioinformatic analysis of data collected from published transcriptomes suggests an increase of *HOXD9 *expression in gliomas and more particularly in glioblastomas as compared to the normal brain tissues and medulloblastomas. Considering the heterogeneity of the samples and methods used, a statistical analysis of these differences was not relevant in these data sets (Figure 1C). However, focusing on a given study was more informative. As per the example of Sun et al 2006 we observe a 2.6-fold increase (*P *value = 2.93E-10)[28]. We also examined the expression of *HOXD9 *mRNA in four glioma cell lines, U87, SK-MG-1, KNS-42 and KNS-81 and found higher expression of *HOXD9 *mRNA compared with that in normal human astrocytes (33-, 32.5-, 52.8-, and 57.5-fold higher, respectively; Figure 1D).

### Immunohistochemical analysis of HOXD9 in gliomas

**Figure 2:**
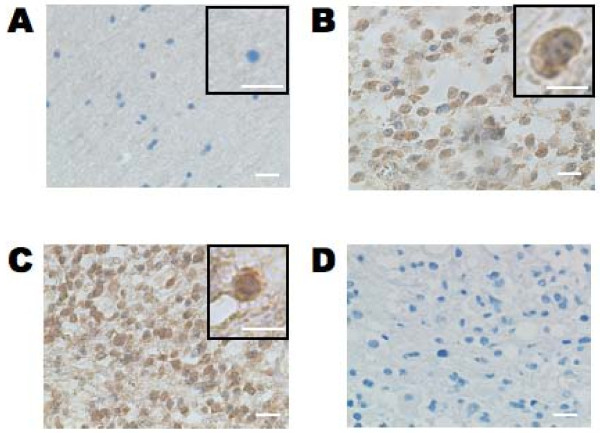
**Immunohistochemical analysis of HOXD9 expression in gliomas and normal brain tissue**. Only a few HOXD9 immunopositive cells were detected in normal tissues from the cerebral cortex in an oligodendroglioma (A). In an anaplastic astrocytoma (B, WHO grade III) and a glioblastoma (C, WHO grade IV), HOXD9 immunopositive cells were observed in both the cytoplasm and the nucleus. (D) No primary antibody control. The sections were counterstained with hematoxylin. The boxed area shows higher magnification images. Scale bar = 25 μm.

Immunohistochemical analysis showed that the number of HOXD9-positive cells was very low in normal cerebral cortex tissue surrounding a surgical biopsy, in agreement with our results from qRT-PCR (Figure 1C and 2A). However, HOXD9-positive cells were observed in both anaplastic astrocytoma (Figure 2B) and GBM tissues (Figure 2C). To examine the antibody specificity, no primary antibody staining was performed (Figure 2D). Furthermore, immunizing peptide adsorption experiments by western blot method (Additional file 1, Figure S1) and immunohistochemical method in kidney tissues (data not shown) showed that weak immunoreactivity, confirming the specificity of the antibody.

In some cells, HOXD9 immunoreactivity was observed in the both cytoplasm and the nucleus (Figure 2B); however, most of the HOXD9 labeling was confined to the nucleus, consistent with its function as a transcription factor (Figure 2C). We also observed no correlation between HOXD9 expression and cell proliferation in gliomas using the MIB-1 index (Table 1).

**Table 1:** Association of HOXD9 expression and MIB-1 index in glioma tissues

*Case no*	*Histology*	*Age (years)*	*Sex*	*MIB-1(%)*^*a*^	*Immunohistochemistry*^*b*^
WHO grade IV					
GB1	Glioblastoma	60	M	40.7	+++
GB2	Glioblastoma	79	F	44.8	+++
GB3	Glioblastoma	72	M	9.1	+
GB4	Glioblastoma	55	F	14.0	+
GB5	Glioblastoma	34	M	8.0	+
GB6	Glioblastoma	28	M	53.0	+++
GB7	Glioblastoma	33	M	8.1	++
GB8	Glioblastoma	55	F	37.2	+++
GB9	Glioblastoma	69	M	30.1	++
GB10	Glioblastoma	38	M	36.4	+
GB11	Glioblastoma	61	F	23.8	+
GB12	Glioblastoma	53	F	11.2	+
GB13	Glioblastoma	60	F	20.4	+
					
WHO grade III					
AA1	Anaplastic astrocytoma	50	F	0.8	+++
AA2	Anaplastic astrocytoma	77	F	2.6	+
AA3	Anaplastic astrocytoma	36	M	1.2	++
AA4	Anaplastic astrocytoma	76	F	34.2	+
AA5	Anaplastic astrocytoma	39	M	2.2	++
AA6	Anaplastic astrocytoma	27	M	5.2	+
AA7	Anaplastic astrocytoma	52	F	19.6	+++
AA8	Anaplastic astrocytoma	45	F	4.0	
AA9	Anaplastic astrocytoma	27	F	7.8	-
AA10	Anaplastic astrocytoma	39	F	3.2	+++
AA11	Anaplastic astrocytoma	9	F	1.6	+
					
WHO grade II					
DA1	Diffuse astrocytoma	37	M	4.5	+
DA2	Diffuse astrocytoma	36	M	3.0	++
DA3	Diffuse astrocytoma	3	M	2.9	++
DA4	Diffuse astrocytoma	50	M	1.	
DA5	Diffuse astrocytoma	10	M	2.4	++

^a ^The proliferating cell index was analyzed in >1000 tumor cells in more than three areas expressing the highest number of MIB-1-positive nuclei. ^b ^The patterns of HOXD9 staining are described as (-) negative or faint staining; (+) positive in less than 30% of tumor cells; (++) positive in less than 70% of tumor cells; (+++) positive in more than 70% of tumor cells. M, male; F, female.

### Gene silencing of *HOXD9 *decreases cell proliferation of glioma U87 cells

**Figure 3:**
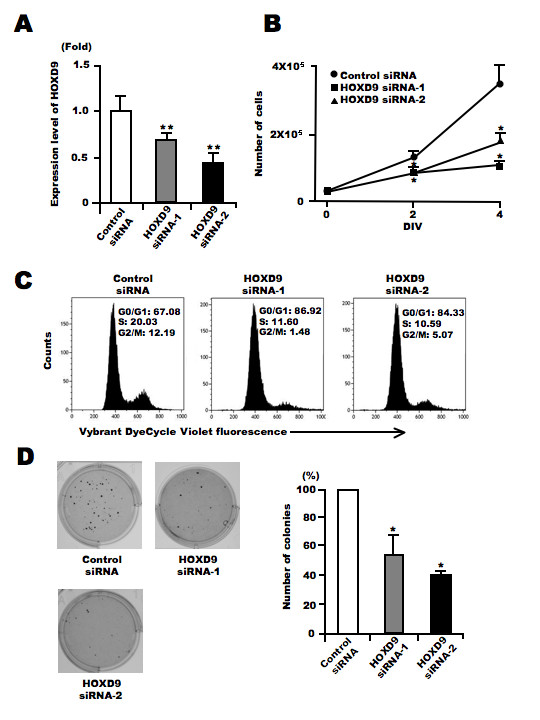
**Silencing of *HOXD9 *gene decreases proliferation of U87 glioma cells**. (A) Analysis of *HOXD9 *gene expression by qRT-PCR 2 days after siRNA transfection. The graphs show the average of two independent experiments. For the graphs, the data are complied from three independent experiments. * *, *P *< 0.01. Error bars indicate ± S.D. (B) Growth suppression of U87 glioma cells after *HOXD9 *siRNA transfection. siRNA-transfected cells were counted by trypan blue exclusion. Data are representative of three independent experiments. *, *P *< 0.05. Error bars indicate ± S.D. (C) Cell cycle alteration by *HOXD9 *siRNA-1 in U87 glioma cells 3 days after siRNA treatment. Results of flow cytometry analysis using Vybrant DyeCycle Violet dye are shown. Data are representative of three independent experiments. (D) 3 weeks after siRNA transfection, colonies stained with MTT [3-(4,5-dimethyl-2-thiazolyl)-2,5-diphenyl-2H-tetrazolium bromide] were counted. For the graphs, the data are complied from three independent experiments. *, *P *< 0.05. Error bars indicate ± S.D.

To investigate the role of *HOXD9 *in tumor cells, we examined changes in U87 glioma cell proliferation in the absence of *HOXD9 *using siRNA. We designed two different siRNAs to reduce the gene expression of *HOXD9 *(Figure 3A). Cell number was counted 2 and 4 days after siRNA transfection into U87 cells. There were fewer cells present after transfection with either of the two *HOXD9 *siRNAs compared with the control siRNA transfected cells (Figure 3B). In addition, the decrease of cell proliferation by *HOXD9 *gene silencing was observed in KNS-42 and KNS-81 glioma cells (Additional file 2, Figure S2). Cell cycle analysis of live cells using Vybrant DyeCycle Violet dye showed that the S-phase and G2-phase cell populations decreased 3 days after *HOXD9 *siRNA transfection in U87 cells (Figure 3C). Furthermore, gene silencing of *HOXD9 *reduced the number of colony formation in U87 glioma cells compared to the control (Figure 3D). These results suggest that *HOXD9 *may be involved in glioma cell proliferation.

### Gene silencing of *HOXD9 *induces apoptosis in U87 glioma cells

**Figure 4:**
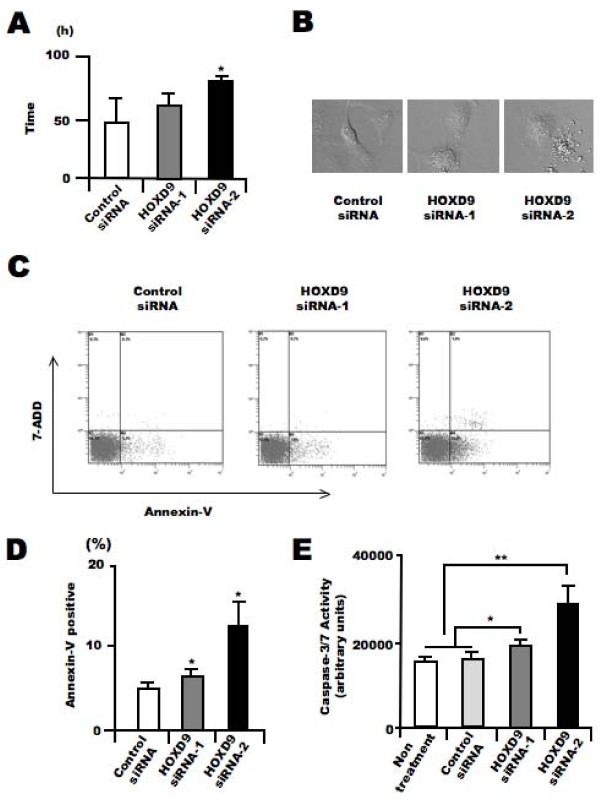
**Silencing of *HOXD9 *gene induces apoptosis in U87 glioma cells**. (A) Average cell division time of siRNA-treated cells was measured for 4 days starting 24 h after transfection (n = 3). *, *P *< 0.05. Error bars indicate ± S.D. (B) Representative images of U87 glioma cells transfected with control or *HOXD9 *siRNA in the time-laps cell imaging analysis of three independent experiments (C) Determination of apoptosis by flow cytometry 3 days after siRNA transfection. Early apoptosis (lower right, Annexin V^+^/7ADD^-^), late apoptosis (upper right, Annexin V^-^/7ADD^+^), necrotic cells (upper left, Annexin V^-^/7ADD^+^), and normal cells (lower left, Annexin V^-^/7-ADD^-^) from a representative experiment at least three independent experiments. (D) Average percentage of Annexin V^+ ^cells. For the graphs, the data are complied from three independent experiments. **, *P *< 0.01. Error bars indicate ± S.D. (E) Analysis of caspase3/7 activity in U87 glioma cells 48 hr after siRNA treatment. For the graphs, the data are complied from three independent experiments. *, *P *< 0.05, **, *P *< 0.01. Error bars indicate ± S.D.

In addition to the analysis of proliferation, we analyzed the effect of *HOXD9 *gene silencing on apoptosis in U87 cells. We evaluated the cell division time and morphological changes in U87 glioma cells after *HOXD9 *siRNA transfection using a time-lapse video microscope system according to our previous study[29]. The average cell division time in *HOXD9 *siRNA-transfected cells was greater than that in the control cells (47.2 ± 18 h for control siRNA vs. 81.1 ± 1.3 h for *HOXD9 *siRNA-2, *p *= 0.03; Figure 4A). Furthermore, many *HOXD9 *cells underwent apoptosis, observed as cell fragmentation (Figure 4B). To confirm whether the morphological changes were the result of apoptotic cell death, we performed flow cytometric analysis using Annexin-V/7-ADD staining. Compared with control cells, the number of Annexin V^+^/7-ADD^- ^cells was significantly higher after *HOXD9 *siRNA transfection on Day 3 (Figure 4C). The total number of Annexin-V^+ ^cells was higher after *HOXD9 *siRNA transfection (Figure 4D). We also measured the activity of caspase 3/7, which is known to promote apoptosis. Two days after *HOXD9 *siRNA transfection, caspase 3/7 activity was induced (Figure 4E).

**Figure 5:**
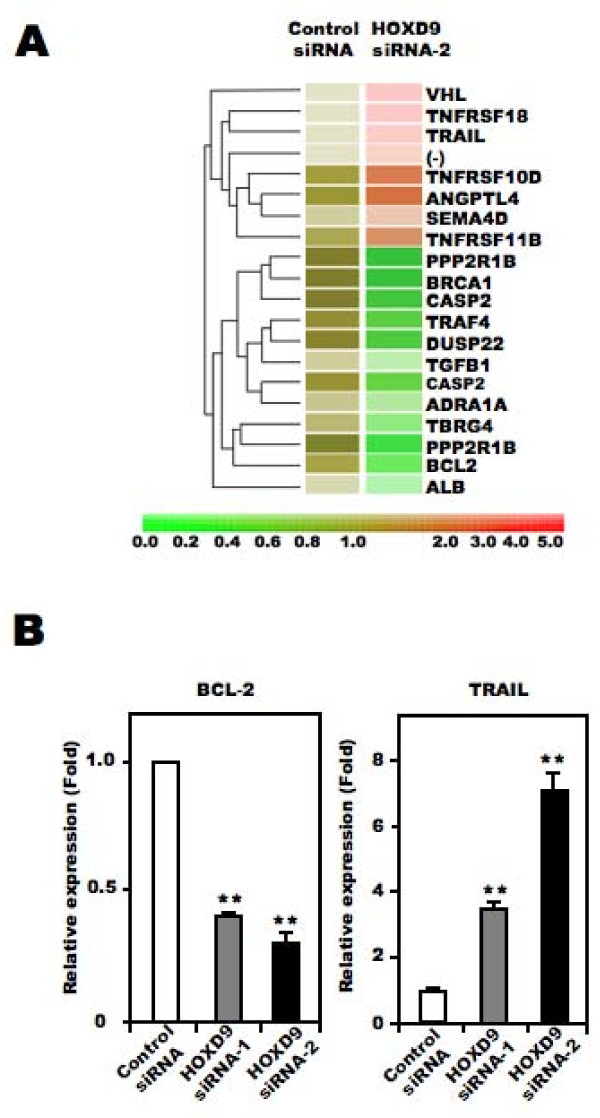
**Effect of *HOXD9 *gene knockdown on the expression of apoptosis- and cell proliferation-related factors**. (A) Gene expression in U87 glioma cells transfected with *HOXD9 *siRNA or control siRNA was determined by microarray analysis. A gene list generated by a Gene Ontology search against "Apoptosis regulator activity" and "Cell growth and/or maintenance" was clustered using a hierarchical method. The red, green, and brown scale represents the expression level of a gene above, below, or equal to the mean expression level for that gene across all samples, respectively. (-) indicates that the gene was not annotated in the NCBI gene bank list. (B) Analysis of *BCL-2 *and *TRAIL *gene expression in U87 glioma cells by qRT-PCR 2 days (*BCL-2*) or 3 days (*TRAIL*) after siRNAs transfection. Each mRNA was normalized against *GAPDH *mRNA and expressed relative to the normalized value for control siRNA-treated cells. For the graphs, the data are complied from three independent experiments. **, *P *< 0.01. Error bars indicate ± S.D.

To identify the molecules involved in cell death associated with *HOXD9 *silencing, we extracted total RNA from control- and *HOXD9*-siRNA transfected cells on Day 2, and subjected them to DNA microarray analysis. Genes that showed a >2-fold change in expression between control siRNA- and *HOXD9 *siRNA-transfected cells were considered to be significantly changed. Figure 5A shows the cluster analysis of selected genes obtained from the Gene Ontology database under the classifications "apoptosis regulator activity" and "cell growth and/or maintenance". Of the listed genes, we focused on *BCL-2*, a known anti-apoptotic factor, and *TRAIL*, a member of the tumor necrosis factor family of cell death-inducing ligands, as candidate apoptosis-related genes in this study. It has been reported that *TRAIL *can induce apoptosis in U87 cells[30]. To confirm the results from the microarray analysis, we performed qRT-PCR analysis to quantify the mRNA level of these two genes. Compared with control siRNA-transfected U87 cells, *BCL-2 *mRNA was lower and *TRAIL *mRNA was higher in *HOXD9 *siRNA treated cells, consistent with the results from the microarray analysis (Figure 5B). Collectively, these results suggest that *HOXD9 *silencing promotes cell death in U87 cells.

### *HOXD9 *is involved in the proliferation of SK-MG-1 SP cells

**Figure 6:**
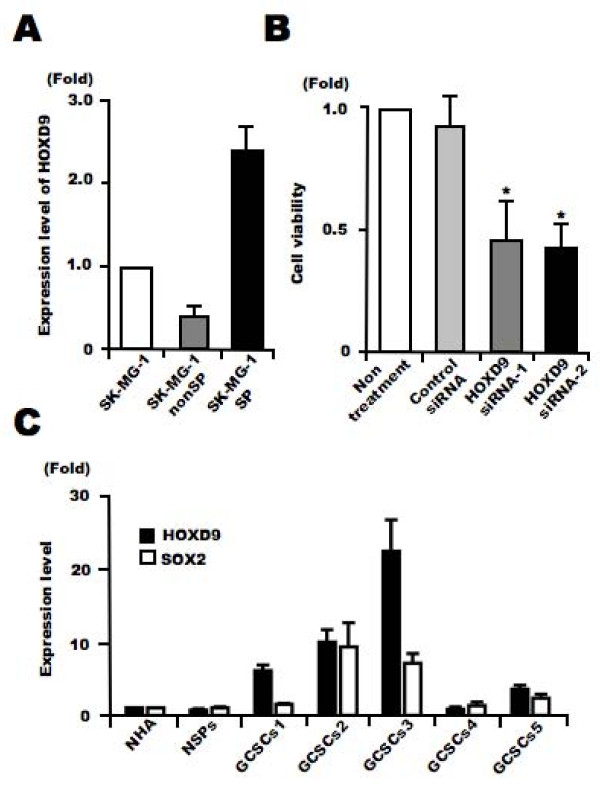
**Analysis of the expression and function of *HOXD9 *in glioma cancer stem cells**. (A) Analysis of *HOXD9 *gene expression in SK-MG-1 SP cells, SK-MG-1 non-SP cells, and total SK-MG-1 glioma cells. The abundance of *HOXD9 *mRNA was normalized to that of *GAPDH *mRNA and expressed relative to the normalized value for SK-MG-1 cells. For the graphs, the data are complied from three independent experiments. Error bar indicates mean ± S.D. (B) Gene knockdown of *HOXD9 *in SK-MG-1 SP cells attenuated cell proliferation. Cell viability was determined 2 days after siRNA treatment. The experiment was repeated twice, with similar results. *, *P *< 0.05. Error bars indicate ± S.D. (C) Expression of the *HOXD9 *and *SOX2 *genes was examined by qRT-PCR in normal human astrocytes (NHA), human NSPCs, and GCSCs cultured from glioma surgical biopsy specimens. The abundance of *HOXD9 *and *SOX2 *mRNA was normalized to that of *GAPDH *and expressed relative to the normalized value for NHA. Data shown are the mean ± S.D. from two independent experiments.

Recently, we found that a side population (SP) of cells in a glioma cell line, SK-MG-1, possesses the properties of glioma stem-like cells[22]. We analyzed the expression levels of *HOXD9 *in SP and non-SP SK-MG-1 cells using qRT-PCR, and found higher expression of *HOXD9 *mRNA in SP cells compared with non-SP cells (Figure 6A). We performed a cell proliferation assay using SP cells transfected with either control or *HOXD9 *siRNA. QRT-PCR confirmed efficient gene silencing of *HOXD9 *in SK-MG-1 SP cells transfected with *HOXD9 *siRNA (data not shown). The viability of SK-MG-1 SP cells was significantly attenuated by *HOXD9 *siRNA compared with control siRNA 48 h after transfection (Figure 6B).

### HOXD9 expression in human GCSCs

We cultured human GCSCs as glioma spheres from glioma surgical specimens and established five cell lines as described in our previous study[10]. *SOX2*, a known neural stem/progenitor cell (NSPC) marker, is also expressed in gliomas and GCSCs[31]. We performed qRT-PCR to examine the expression of *HOXD9 *and *SOX2 *in GCSCs compared with normal primary astrocytes and NSPCs derived from fetal brain and cultured as neurospheres[32]. The expression of *SOX2 *and HOXD9 in GCSCs was higher than that in normal astrocytes and NSPCs. Also, *HOXD9 *expression was higher than *SOX2 *expression in some GCSCs (Figure 6C).

## Discussion

This is the first study examining the function of *HOXD9 *in gliomas. We found that *HOXD9 *was more highly expressed in gliomas and GCSCs and that gene silencing of *HOXD9 *reduced the proliferation of both glioma cells and glioma cancer stem-like cell population.

The expression of homeobox family genes is generally restricted during embryogenesis. Recently, it was reported that *HOXD9 *is expressed in murine neural tubes and neural crest cells during development[33]. In this study, we observed the high expression of *HOXD9 *in normal adult human kidney and testis. The misexpression of homeobox transcription factor genes has also been reported in cancer tissues; for example, *HOXA1 *and *Six1 *transform mammary epithelial cells[34,35], and *Msx1 *and *Cdx *transform myoblasts[36] and intestinal epithelial cells[37], respectively. Although the mechanisms underlying the misexpression of homeobox transcription factor genes in cancer remain elusive, the deregulation of non-coding RNA expression and/or changes in the methylation status of the promoters may be involved. Recently, it has been reported that non-coding RNA residing in the *HOXC *locus could act in *trans *to regulate transcription of the *HOXD *locus with the Polycomb-repressive complex 2 (*PRC2*)[38]. *HOXD11 *and *HOXD12 *are regulated by the Polycomb group proteins during embryonic stem cell differentiation[39]. We performed bisulfate sequencing to compare the methylation status of the *HOXD9 *promoter in U87 cells compared with normal human T cells and NSPCs. Hypermethylation of CpG islands was observed in the *HOXD9 *promoter region in U87 cells compared to T cells and NSPCs when *HOXD9 *gene expression was high (Additional file 3, Figure S3). This relationship between gene expression and methylation status has also been observed in *HOXB *family genes in small-cell lung cancer[40]. Hypermethylation of CpG islands in promoter regions has been reported for many genes, including the *HOXC *and *HOXD *cluster genes associated with *HOXD9 *in human astrocytomas[39,41]. It is difficult to understand why hypermethylation correlates with increased rather than decreased gene expression. In the future, it may be important to evaluate the methylation status of *HOXD9 *in the whole genome, as well as histone modification in gliomas and GCSCs compared with normal brain and NSPCs using the tiling array system and/or a next-generation sequencer. Thus, the analyses of epigenetic regulation of *HOXD9 *gene expression in gliomas and GCSCs related to Polycomb proteins and non-coding RNA will be an important issue in the near future.

We showed that gene knockdown of *HOXD9 *reduces the proliferation of U87, KNS-42, and KNS-81 glioma cells and glioma cancer stem-like cells; SK-MG-1 SP cells. So far, *HOXD9 *is reported to be involved in the regulation of cell proliferation in rheumatoid arthritis[42] and carcinogenesis[43], indicating that *HOXD9 *may contribute to cell proliferation in *HOXD9*-expressing cells in gliomas including GCSCs In our preliminary experiment, transiently over-expressed *HOXD9 *increased the S-phase cell population of U87 cells in cell cycle analysis (data not shown). In this study, gene silencing of *HOXD9 *induced apoptosis and reduced the expression of *BCL-2 *in glioma cells, indicating that *HOXD9 *may support the cell survival. Furthermore, immunohistochemical studies showed no apparent correlation between HOXD9 expression level and WHO grade or MIB-1 index, suggesting that HOXD9 may be expressed in primitive cancer cell populations, including GCSCs *in vivo*. As for the upstream factors of *HOXD9*, it has been reported that *HOXD9 *expression is induced by *Wnt *signaling[33], which is a maintenance factor for neural stem cells[44] and neural crest cells[45], suggesting that *HOXD9 *may act as a maintenance or survival factor for GCSCs undercontrol of *Wnt *signaling. *HOXB1 *also supports the maintenance and expansion of neural progenitor cells[46], and *HOXB4 *may expand hematopoietic stem cells[47]. Taken together, these results indicate that some homeobox proteins including *HOXD9 *may contribute to cancer stem cell maintenance in addition to the cell proliferation and/or survival.

In the present study, we demonstrated that *HOXD9 *was highly expressed in glioma cells and GCSCs cultured from patient specimens compared with human NSPCs and astrocytes. To date, drug development for targeting cancer stem cells is an important issue in cancer therapy[48]. Therefore, it is very important for this purpose to find targets expressed in cancer stem cells with a high specificity. In this point, *HOXD9 *may be an ideal therapeutic target for the treatment of gliomas because the expression in NSPCs and astrocytes is lower than GCSCs, suggesting that *HOXD9 *targeted therapy may have a therapeutic index. In fact, knockdown of *HOXD9 *decreased the proliferation of glioma cancer stem-like cells *in vitro*, supporting the idea. Recently, it has been reported that *HOXA9 *decreases apoptosis and increases cell proliferation in glioma cells by epigenetic control[49] and the expression of *HOXA10 *in GBM-derived spheres[50]. Thus, it may be intriguing to study the detailed analysis of *HOX *families in GCSCs in the future for the development of drug discovery targeted for GCSCs.

## Conclusions

*HOXD9 *may be useful as a marker for glioma and GCSCs, and therapies targeting *HOXD9 *should be considered for further development.

## Competing interests

The authors declare that they have no competing interests.

## Authors' contributions

Conceived and designed the experiments: MT^1 ^SO MT^2^. Performed the experiments: MT^1 ^SO YO RF AM CT. Analyzed the data: MT^1 ^SO. Contributed reagents/materials/analysis tools: KY TK HS HC YM HO YK. Wrote the paper: MT^1 ^SO HC MT^2 ^(MT^1^, M. Tabuse; MT^2^, M. Toda). All authors read and approved the final manuscript.

## Supplementary Material

Additional file 1 **Figure S1. Peptide pre-absorption analysis of Western blot**. The anti-HOXD9 antibody was pre-incubated with blocking peptide (antigen) by 1:5 Weight ratio at 4°C overnight. Immunizing peptide adsorption showed weak immunoreactivity towards over-expressed HOXD9 in 293T cells. Click here for file

Additional file 2 **Figure S2. Silencing of *HOXD9 *gene decreases proliferation of KNS-42 and KNS-81 glioma cells**. (A) Analysis of *HOXD9 *gene expression by qRT-PCR analysis 2 days after siRNA transfection in KNS-42 and KNS-81 glioma cells. (B) Gene knockdown of *HOXD9 *in in KNS-42 and KNS-81cells attenuated cell proliferation. Cell viability was determined 2 days after siRNA treatment. For the graphs, the data are complied from three independent experiments. *, *P *< 0.05, **, *P *< 0.01. Error bars indicate ± S.D. Click here for file

Additional file 3 **Figure S3. Analysis of CpG methylation**. (A) A schematic representation of the previously described restriction landmark genomic scanning (RLGS) clone[16] and the position of the CpG islands within the promoter region of the *HOXD9 *gene. (B) Methylation maps derived from bisulfate sequencing analysis of human T cells, NSPCs, and U87 glioma cells. ●, methylated; ○, unmethylated. Click here for file
