# Supplementary figures and images for: Functional analysis of HOXD9 in human gliomas and glioma cancer stem cells
Source: Mol Cancer. 2011 May 22;10:60. doi: 10.1186/1476-4598-10-60 (PMC3118386; doi:10.1186/1476-4598-10-60)

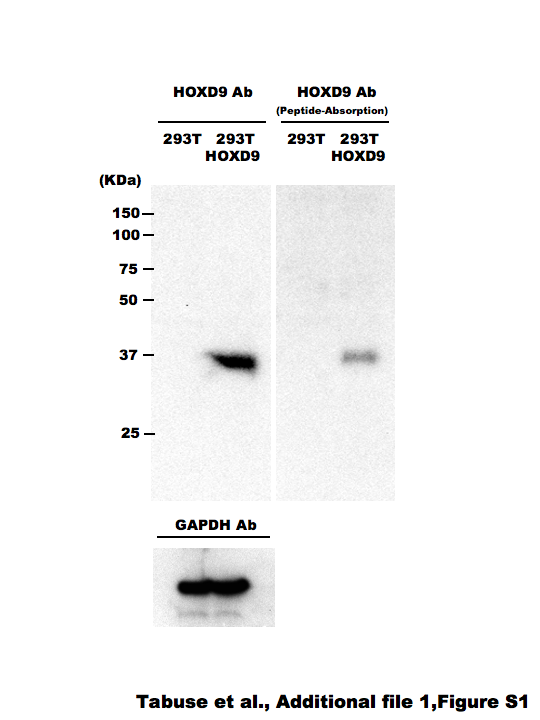

Supplement: Additional file 1 — Figure S1. Peptide pre-absorption analysis of Western blot. The anti-HOXD9 antibody was pre-incubated with blocking peptide (antigen) by 1:5 Weight ratio at 4°C overnight. Immunizing peptide adsorption showed weak immunoreactivity towards over-expressed HOXD9 in 293T cells. [file 1476-4598-10-60-S1.TIFF]

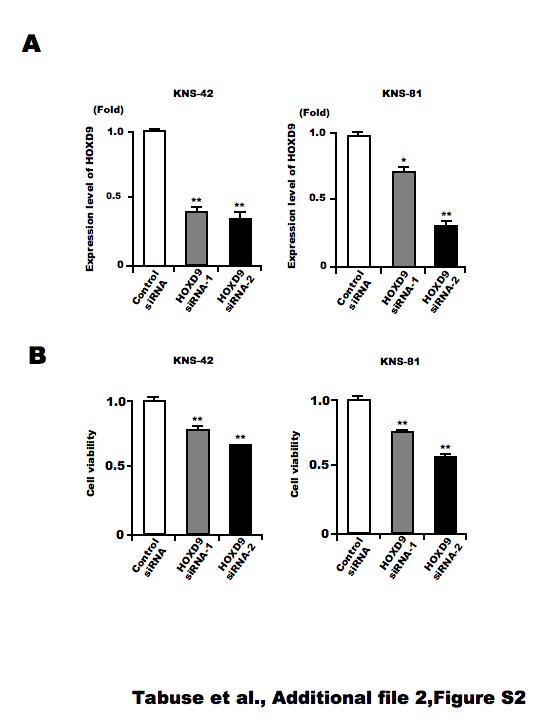

Supplement: Additional file 2 — Figure S2. Silencing of HOXD9 gene decreases proliferation of KNS-42 and KNS-81 glioma cells. (A) Analysis of HOXD9 gene expression by qRT-PCR analysis 2 days after siRNA transfection in KNS-42 and KNS-81 glioma cells. (B) Gene knockdown of HOXD9 in in KNS-42 and KNS-81cells attenuated cell proliferation. Cell viability was determined 2 days after siRNA treatment. For the graphs, the data are complied from three independent experiments. *, P < 0.05, **, P < 0.01. Error bars indicate ± S.D. [file 1476-4598-10-60-S2.TIFF]

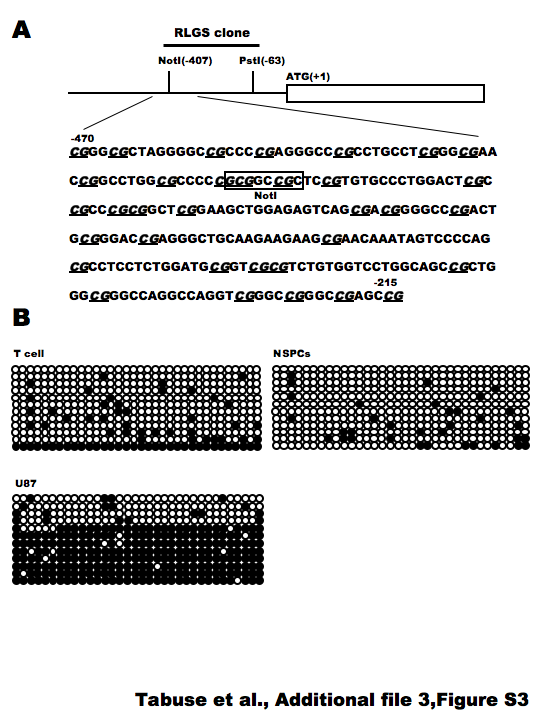

Supplement: Additional file 3 — Figure S3. Analysis of CpG methylation. (A) A schematic representation of the previously described restriction landmark genomic scanning (RLGS) clone[16] and the position of the CpG islands within the promoter region of the HOXD9 gene. (B) Methylation maps derived from bisulfate sequencing analysis of human T cells, NSPCs, and U87 glioma cells. ●, methylated; ○, unmethylated. [file 1476-4598-10-60-S3.TIFF]
